# The Need and Potential of Biosensors to Detect Dioxins and Dioxin-Like Polychlorinated Biphenyls along the Milk, Eggs and Meat Food Chain

**DOI:** 10.3390/s111211692

**Published:** 2011-12-15

**Authors:** Jeerasak Chobtang, Imke J. M. de Boer, Ron L. A. P. Hoogenboom, Willem Haasnoot, Aize Kijlstra, Bastiaan G. Meerburg

**Affiliations:** 1 Animal Production Systems Group, Wageningen University, P.O. Box 338, 6700 AH Wageningen, The Netherlands; E-Mails: jeerasak_lim@hotmail.com (J.C.); imke.deboer@wur.nl (I.J.M.B.); 2 Livestock Research, Wageningen University and Research Centre, P.O. Box 65, 8200 AB Lelystad, The Netherlands; E-Mail: aize.kijlstra@wur.nl (A.K.); 3 RIKILT Institute of Food Safety, Wageningen University and Research Centre, P.O. Box 230, 6700 AE Wageningen, The Netherlands; E-Mails: ron.hoogenboom@wur.nl (R.L.A.P.H.); willem.haasnoot@wur.nl (W.H.); 4 Eye Research Institute Maastricht, Department of Ophthalmology, University Hospital Maastricht, P.O. Box 5800, 6202 AZ Maastricht, The Netherlands

**Keywords:** dioxins, biosensor, polychlorinated biphenyls, food chain

## Abstract

Dioxins and dioxin-like polychlorinated biphenyls (DL-PCBs) are hazardous toxic, ubiquitous and persistent chemical compounds, which can enter the food chain and accumulate up to higher trophic levels. Their determination requires sophisticated methods, expensive facilities and instruments, well-trained personnel and expensive chemical reagents. Ideally, real-time monitoring using rapid detection methods should be applied to detect possible contamination along the food chain in order to prevent human exposure. Sensor technology may be promising in this respect. This review gives the state of the art for detecting possible contamination with dioxins and DL-PCBs along the food chain of animal-source foods. The main detection methods applied (*i.e*., high resolution gas-chromatography combined with high resolution mass-spectrometry (HRGC/HRMS) and the chemical activated luciferase gene expression method (CALUX bioassay)), each have their limitations. Biosensors for detecting dioxins and related compounds, although still under development, show potential to overcome these limitations. Immunosensors and biomimetic-based biosensors potentially offer increased selectivity and sensitivity for dioxin and DL-PCB detection, while whole cell-based biosensors present interpretable biological results. The main shortcoming of current biosensors, however, is their detection level: this may be insufficient as limits for dioxins and DL-PCBs for food and feedstuffs are in pg per gram level. In addition, these contaminants are normally present in fat, a difficult matrix for biosensor detection. Therefore, simple and efficient extraction and clean-up procedures are required which may enable biosensors to detect dioxins and DL-PCBs contamination along the food chain.

## 1. Introduction

Polychlorinated dibenzo-p-dioxins (PCDDs) and dibenzofurans (PCDFs) are a group of chemical compounds, collectively known as “dioxins”, produced by chemical processes, combustion and waste incineration, involving chlorine [1]. In particular the seventeen 2,3,7,8-chlorinated PCDD/Fs are considered as a a major threat. A major problem of these compounds is their persistence in the environment. They can enter the food chain at the lowest trophic level of the production chain and accumulate up along the chain. Their persistence originates in their resistance to metabolic degradation and their lipophilic character [2]. Properties of dioxins have been reviewed extensively elsewhere [3–7].

**Figure 1.**
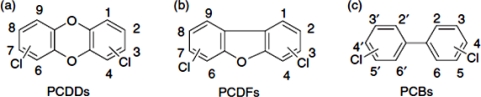
Basic chemical structure of polychlorinated dibenzo-p-dioxins (**a**), polychlorinated dibenzofurans (**b**) and polychlorinated biphenyls (**c**) [3].

In addition to dioxins, polychlorinated biphenyls (PCBs) are a group of chemical compounds that have been produced for industrial purposes [8]. Although the commercial production and use of PCBs was banned since the late 1970s [7], they are ubiquitous and frequently encountered in the environment, wildlife tissue and the food chain. Of the 209 different PCBs, 12 congeners show dioxin-like toxicity (DL-PCBs). These tetra- to heptachlorinated non- or mono-*ortho* PCBs share the planar structure and metabolic persistence of dioxins. Due to their toxicity and strict measures to reduce human exposure, dioxins and DL-PCBs are a major threat for production of safe feed and food [9]. Figure 1 illustrates the chemical structure of dioxins and DL-PCBs.

In addition to the DL-PCBs, there is the much larger group of non-dioxin-like PCBs. In practice only a limited number of these NDL-PCBs are determined as indicator compounds for the group (e.g., PCB’s 28, 52, 101, 138, 153 and 180). In most countries limits were set in the past for these so-called indicator PCBs. The EU decided to harmonize these limits and new limits will become effective in 2012. In general these limits are an order of magnitude higher than those for dioxins and DL-PCBs, also meaning that their detection will be easier to achieve. For milk e.g., the limit will be 40 ng/g fat as compared to 3 and 6 pg TEQ/g fat for dioxins and the sum of dioxins and DL-PCBs.

Since the Belgian dioxin incident in 1999, which strongly affected consumer confidence in foodstuffs and caused huge financial losses [10], there has been an increased consumer awareness of the danger caused by dioxins and DL-PCBs contamination. In December 2010, a dioxin contamination in animal-source food including eggs, poultry meat and pork was detected in Germany. The consequence was not only the destruction of thousands of chickens and hundreds of thousands of eggs but also a dramatic drop in egg consumption and the export of those products [11]. Alcoser *et al*. [12] quantified the financial effects of a possible dioxin contamination in the Dutch milk chain. They conclude that in order to minimize financial impact, contamination should be detected in the first stage of the production chain. Thus, precise, accurate and timely surveillance programs are helpful in order to minimize financial impacts of a potential contamination. Quantification of the concentration of dioxins and DL-PCBs is however challenging. This is because the limits are very low and different for a variety of different sample matrices. Furthermore, many other organic contaminants can potentially interfere in the measurement [13]. The golden standard for analysis of dioxins and DL-PCBs is high resolution gas-chromatography combined with high resolution mass-spectrometry (HRGC-HRMS). However, this method is quite expensive and has a limited sample throughput. Therefore a number of screening assays has been developed, including bioassays such as the Chemical Activated LUciferase gene eXpression (CALUX) method, that was successfully established for screening dioxins and DL-PCBs [14]. Such methods, however, still require additional sophisticated preparations, instruments and techniques, and a dedicated laboratory. Also, they require a number of days before the result is obtained. There is an urgent need for technology that can detect the contamination more rapidly, but is still accurate and reliable. For this purpose, sensor technology might prove useful. Such technology was successfully developed for several domains, including health care [15–17], environmental security monitoring [18–21] and food safety screening [22–25]. However, in case of dioxins and DL-PCBs, there is a lack of studies on sensor technology, although they (especially biosensors) could be promising technologies. This review aims at describing the potential of biosensors against the background of dioxins and DL-PCB contamination in the food chains of milk, eggs and meat. State of the art of different technologies, biosensor technology in particular, for detection of dioxins and DL-PCBs will be discussed, based on the following criteria, *i.e*., validity, simplicity, sensitivity, relevance and feasibility. Finally, we will address the potential of biosensors for detection of dioxins and DL-PCB contamination throughout the food chain.

## 2. Dioxin and DL-PCB Contamination Risk in the Milk, Eggs and Meat Food Chain

Among the different congeners of dioxins and DL-PCBs, the most toxic congener is 2,3,7,8-tetrachlorinated dibenzo-*p*-dioxin (TCDD) [6]. Other dioxins and DL-PCBs show different toxic potencies. In order to deal with this difference in potencies, the so-called toxic equivalents (TEQ) principle was introduced [26]. In the past decennia, this principle has frequently been re-evaluated [6,27]. Based on results mainly from *in vivo* experimental toxicity studies, each congener is assigned a relative potency factor or TEF, expressing the toxicity in comparison to TCDD (assigned TEF of 1). When analyzing a sample, the levels of individual congeners are multiplied by TEF values and summed to arrive at a TEQ-level [6].

TCDD is considered to be a human carcinogen [28]. Besides being carcinogenic, a broad spectrum of adverse effects of dioxins and DL-PCBs has been reported in animals and humans [5,29–31], including declining sperm counts [32], immunosuppression, developmental and reproductive disorders, endocrine disruptions and skin disorders [5,29,33]. The main mode of action of these toxins is mediated through an interaction of these compounds with the intracellular aryl hydrocarbon receptor (AhR) [3,34]. This can result in an alteration of gene expression including genes involved in the metabolism of various compounds including endogenous hormones. However, it is important to realize that also other compounds may bind to AhR, including compounds naturally occurring in plants [35–37] but also a number of the polyaromatic hydrocarbons (PAHs) [38,39].

Based on the adverse effects in animals during animal experiments, the Scientific Committee on Food established a Tolerable Weekly Intake (TWI) level of 14 pg TEQ/kg bw/week. This limit should prevent that body levels in consumers will eventually reach a critical level. It was shown that part of the population still exceeds this TWI. The consumption of contaminated animal-source food appears the main source of dioxin and DL-PCB exposure in humans [40,41]. Therefore, the maximum level of these compounds in meat and meat products, milk, eggs and animal fat is strictly regulated by the European Union (Table 1) to reduce the risk of human exposure [42]. Since dioxins and DL-PCBs accumulate in fat, limits in food are lipid-based, except for fish, where the lipid content is highly variable, also within one species. Limits will change in 2012 based on the use of the TEFs from 2005.

In general, oral exposure is the main route of dioxin and PCB contamination for both animals and humans. After absorption via the gastrointestinal tract, some congeners are metabolized into non-toxic compounds and excreted [27,43]. In particular the 2,3,7,8-substituted dioxins and higher chlorinated PCBs are more resistant to degradation and stored in tissues and organs [27,44] and only to some extent excreted as entire congeners or metabolites through feces and urine [27,43,44]. Elimination pathways of dioxins and DL-PCBs depend on several factors, such as animal species, sex, age and physiological stage of production [45–47]. Lactating animals, for instance, excrete part of these toxins via milk fat with carry-over rates of 34 to 60% dependent on the degree of chlorination *i.e*., the higher numbers of chlorine molecule (7 or 8) congeners are transferred with lower carry-over rates than the lower chlorinated (4, 5 or 6) ones [48]. Laying hens eliminate them through egg production with carry-over rates ranging from 4 to 76%, again dependent on the site and degree of chlorination [49].

Since dioxins can enter the animal food chain in different ways, in this study, we applied a “cradle-to-farm-gate” approach to milk, egg and meat production in order to determine possible routes in which dioxins and DL-PCBs enter the food chain. Contamination due to post-farm processes, therefore, is not considered but based on the various incidents, this route seems less important. Dioxins and DL-PCBs mainly enter the food chain by oral ingestion of contaminated substances, such as compound feed or feed supplements, roughages, water, soil, and worms or insects. The level of exposure to these compounds in animal production systems is also affected by farm management. Nowadays, consumers pay more attention to animal production systems where food is produced in an animal-friendly way [50]. An example is a system which allows an animal to use an outdoor area to graze, roam or scavenge. However, in this system, the animals have a higher chance of becoming exposed to contaminants from the environment [50–56].

**Figure 2.**
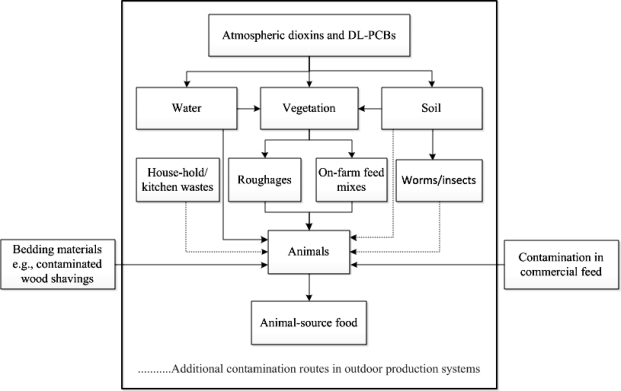
Possible contamination routes along the food chain of animal-source food.

Figure 2 depicts possible contamination routes of dioxins and PCBs along the food chain of animal-source food such as milk, eggs and meat. Because dioxins and PCBs may enter the food chain through several pathways, the magnitude of contamination differs depending on the frequency of exposure and levels of contaminants in each of these pathways. Earlier studies have shown that roughages in general contain higher levels of dioxins than commercial compound feed [57]. In Table 2 the contamination routes for different production systems (milk, eggs and meat) are further elaborated. Different contamination risks were assessed and scored. Ingestion of contaminated soil, worms, insects and roughages are likely to be the major cause of contamination. In addition, dioxins and PCBs can spread over the agricultural environment by the influence of climatic factors [58,59]. Through flooding for instance, contaminated particles or sediment can be transported to areas that are not yet contaminated, such as pastures [47]. As a result, also climate change can play a role as a factor of dioxin and PCB spreading over non-contaminated areas [46].

Dioxins can be potentially emitted from fires, including those from forest, bush and grassland, to the atmosphere [60–63]. It has been documented that dioxin concentration in forest soil and ash were elevated immediately after a forest fire [60]. Moreover, house-hold waste incineration can cause a considerable amount of these toxins [64,65] which can spread through fumes. Contaminated fumes might be transported over longer distances and may eventually be deposited onto the soil or plants. If these are ingested by the animals, this may cause dioxin contamination. Of course, risks are lower when the distance from the source increases. It was demonstrated that the dioxin concentration in ambient air and soil samples taken near a solid waste incinerator were much higher than in samples taken at larger distance [66]. Furthermore, burning waste on or next to the yard close to laying hen housing might be a source of dioxin contamination of home-produced eggs [67]. Occasionally also industrial fires may be a source of dioxins and investigating whether this is the case may prevent future problems.

Interestingly, about 38% of dioxin exposure in the Dutch population can be attributed to the consumption of milk and milk products [40], primarily due to the high consumption of these products. It has been reported that feed is the main source of dioxins and DL-PCBs contamination of cow’s milk [68]. In general, levels in feed are low but sometimes contaminated feed may be responsible for levels exceeding the maximum limit. In the Netherlands, for example, potato peels containing kaolinic clay with high levels of dioxins resulted in elevated levels of dioxins and DL-PCBs in milk [69]. In Italy, an increased level of dioxins and DL-PCBs has been observed in milk from dairy cows that grazed on contaminated pastures in the vicinity of a factory that previously produced PCBs [70]. Similar may be the case in the vicinity of municipal waste incinerators, although many of these companies have improved their process and are under strict control.

The presence of dioxins and DL-PCBs in milk mainly originates from the consumption of roughage since this is the main feed source for dairy cows (Table 2). In addition, involuntary ingestion of contaminated soil is also a major contributor of dioxin and DL-PCB contamination. Dairy cows can ingest between 1 to 10 kg of soil a day when grazing, depending on herd and pasture management [54]. As mentioned earlier, the excretion via milk fat is an important pathway for elimination of dioxins and PCBs for lactating cows [71]. Furthermore, fat mobilization during lactation of the animal also influences dioxin and DL-PCB concentrations in milk. During early lactation, a cow can be in a negative energy balance, implying that she utilizes energy from body fat. This results in release of dioxins and PCBs that were stored in this fat, and hence, to elevated levels of contaminants in milk [72].

Approximately 5% of total daily exposure of the Dutch population to dioxins and DL-PCBs has been estimated to originate from egg consumption [40]. The increased demand of free-range and organic eggs in European countries (the result of increasing consumer interest in animal welfare) may lead to an increase of the exposure of consumers to dioxins and DL-PCBs, since the levels of these compounds in these types of production systems may be higher than in conventional egg production systems [51,53,55,73]. Recent measures and strict control is likely to have resulted in a decrease of the levels in these types of eggs. At the same time farmers still encounter problems to meet the required safety limits. For the same reason, also home-produced eggs in Belgium [56,74,75] and Italy [67] showed higher levels of dioxins and DL-PCBs than eggs from commercial systems. In addition to soil, domestic waste might be one of the contributors of dioxins and DL-PCB contamination in such eggs [75]. These phenomena represent the contradictory concept between food safety and animal-friendly production systems.

Application of wood shavings and sawdust prepared from wood, which has been treated with pentachlorophenol (PCP) as animal bedding material, may also cause increased levels of dioxins in food products [76]. It was found that this was a source of contamination in different foods from animal origin: milk [47], eggs [77] and beef [78]. For example, some congeners of dioxins were detected in eggs laid by laying hens reared on PCP contaminated wood shavings [77]. Another incident was caused by PCP-contaminated sawdust used as a carrier for choline chloride, a feed additive, thus contaminating animal feed [76].

Higher TEQ-values were observed in meat from outdoor than from indoor production systems [79]. Ingestion of contaminated soil and roughages were identified as the main causes of these elevated levels. Beef can also be considered as an important pathway of dioxin and DL-PCB exposure of humans [40,80], where beef originating from extensive production systems may contain higher levels of dioxins and DL-PCBs than beef from intensive production systems [81]. Similar findings were observed for pigs reared in outdoor systems [82]. This may be due to the fact that in extensive systems animals are allowed to graze in pastures that can be contaminated with toxins from atmospheric deposition. Moreover, as is the case of dairy cattle, beef cattle may also involuntarily ingest contaminated soil. Contrary to dairy cattle, dioxins are not excreted through the milk and accumulate in the fat. Furthermore, it was found that meat from calves that relied on milk from suckler cows until slaughter contained higher levels of dioxins and DL-PCBs than that of the animals that were fed with milk replacer [57,81]. This might be a consequence of mobilization of dioxins and DL-PCBs in the cow’s tissues and the transfer into milk fat [81].

The use of contaminated feed ingredients resulted in elevated levels of dioxins and PCBs in pork in Belgium [83], Chile [84], the Netherlands [85], Germany [85–87] and Ireland [9]. Spitaler *et al*. [88] found that the concentration of dioxins and PCBs in pig meat were significantly increased when pigs ingested a higher quantity of contaminants. However, if the contaminated feed is replaced with clean feed, the concentration in finishing pigs will decrease rapidly again. This is primarily caused by a dilution effect: due to their fast growth the body mass/fat ratio will be influenced [89].

Monitoring programs for early detection of the contaminants in all production stages may be the most promising approach to limit dioxins and DL-PCB contamination in animal-source food. The use of critical control points (especially of those that are marked as high risk in Table 2) might eventually reduce human exposure. To accomplish this purpose, fast, accurate and reliable technologies for detecting dioxins and DL-PCBs are needed. Since producers pay the testing, cost of the monitoring programs is an important issue.

Considering the contamination of dioxins and DL-PCBs along the milk, eggs and meat food chain, it could be concluded that the avoidance of contaminated substances or the use of certified dioxin-free feed ingredients are the best strategies to reduce or eliminate the contamination in food from animal origin. Except the accidental addition of contaminated raw materials into animal feed, soil is likely to be a main contributor of dioxins and DL-PCBs. From the soil, the contaminants can be distributed further to vegetation and soil organisms. Additional critical points should therefore be monitored when animal-source foods are produced by outdoor production systems.

## 3. Assays for Detecting Dioxins and DL-PCBs in the Food Chain

Surveillance and monitoring of dioxins and DL-PCBs in feed and food are a good strategy to evaluate the risk for animal and human exposure. Moreover, the contamination level in feed and food can be used for strategic decisions to reduce contamination in the food chain, e.g., through the identification and elimination of specific sources. Sophisticated technologies are needed to detect dioxins and PCBs throughout the food chain. Current detection methods each have their own advantages and limitations. Here, we will describe the methods that are or might be used to determine dioxins and DL-PCBs and classify them into three categories: chemical analysis, bioassays and sensor technology.

### 3.1. Chemical Analysis

Due to very low regulatory limits set for dioxins and DL-PCBs in food and feed, and the need to analyze 29 different congeners, it is very challenging to detect them and quantify their concentration in a reliable manner. The routine method used for quantifying the concentrations of different congeners of dioxins and DL-PCBs is the high resolution gas-chromatography coupled to high resolution mass-spectrometry (HRGC-HRMS). This method requires a sophisticated clean-up step to separate the compounds from fat and other contaminants that may interfere with the analysis. The HRGC-HRMS technique is at present the only accepted method that can quantify the concentration of different congeners of dioxins and DL-PCBs at very low detection limits [13]. The instruction to use this method was established by the United States Environmental Protection Agency, EPA [90] (see Reiner [13,91]). The analysis, however, is costly because of the need for sophisticated instruments, the availability of well-trained staff and the use of expensive chemicals and standards. The analysis of non-dioxin-like PCBs has been extensively reviewed elsewhere [7,92]. Recent developments have improved the clean-up procedure for samples and reduced the time of analysis to less than one day, of course depending on the number of samples to be analyzed. The instruments and chemicals required for the rapid clean-up of samples further increase the costs of analysis.

In general, chemical analysis offers excellent sensitivity for measuring dioxin levels in food, because of its very low detection limits. The main drawback of chemical analysis based on HRGC-HRMS is the lack of information about potential other dioxin-like compounds, like the brominated or mixed halogenated dioxins [93]. At present it is unclear what the contribution of these compounds is to the total amount of dioxin-like compounds.

### 3.2. Bioassays

Several bioassays, which commonly involve living organisms or tissues that sense toxic substances, have been established for detection and quantification of dioxin and PCB contamination in the food chain or its environment. These bioassays quantify the magnitude of contamination by the expression of responsive reporter genes. Gene expression is mediated by binding of ligands to the intracellular aryl hydrocarbon receptor (AhR) [94]. The reporter gene can be a gene already present in the organism, like the one encoding for cytochrome P450 1A enzymes. Increased expression can be detected by adding a specific substrate, like ethoxyresorufin, to the cells after exposure to dioxins and DL-PCBs. This so-called ethoxyresorufin O-deethylase (EROD) activity has been a suitable biomarker for detecting dioxins [95]. More recent developments are based on the introduction of a specific reporter gene into the cells, like luciferase or green fluorescent protein [14,96]. In general, these assays measure the specific production of proteins by transfected cells that are activated when they are exposed to dioxins or dioxin-like compounds, in accordance with the concentration of contaminants. Among such assays, the CALUX bioassays are already extensively used in monitoring programs [14,97–100]. An important feature of these cell-based tests is the fact that the relative potency of the different dioxins and DL-PCBs correlates well with the established TEF-values. However, the relation is not absolute and some correction to account for this deviation is required when testing samples for compliance with the regulatory limits. The CALUX bioassay was applied for the first time during a dioxin contamination of citrus pulp pellets from Brazil in 1998, and subsequently during the dioxin incident in Belgium in 1999, where contaminated PCB transformer oil was mixed with feed fat and entered the food chain [14,101,102].

As the CALUX bioassay measures the response following ligand binding to the intracellular aryl hydrocarbon receptor, many other compounds including both natural and synthetic compounds can interfere with the assay. Superinduction of the aryl hydrocarbon receptor (AhR), caused by other AhR active substances such as polybrominated compounds, or activation of protein kinase C, resulted in an inaccurate overestimation of dioxins [103]. Moreover, it was recently found that natural AhR ligands from foods or supplements can give a positive result in the CALUX bioassay [104]. Furthermore, not only the presence of AhR agonists [97], but also the existence of AhR antagonists [105], can in theory affect the accuracy and precision of the CALUX bioassay. Whether such effects are relevant depends both on the co-occurrence of such compounds and their fate during the clean-up step.

Several laboratories, therefore, have investigated methods to clean-up the sample thus improving the accuracy and precision of the CALUX method. Such a step is also required to separate the dioxins and DL-PCBs from the fat, which is essential for most types of samples. Hoogenboom *et al.* [14] demonstrated that an acid-silica clean-up method is well suited for this purpose. This relatively simple clean-up method markedly improves the specificity for dioxins and DL-PCBs. Jeong *et al*. [106] compared the efficiency of a biological assay and HRGC-HRMS for determination of dioxins and DL-PCBs in meat and animal feed. Their results demonstrated that the bioassay correlated well with the HRGC-HRMS method. Chao *et al*. [107] developed a fast clean-up method for determining the contamination of dioxins and DL-PCBs in soil and sediment samples and used the extracts in the CALUX bioassay. These authors found that the accuracy and precision of the assay were improved following sample pretreatment. Also, Stypula-Trebas *et al*. [108] developed CALUX extraction and clean-up procedures for determination of dioxins in feed samples. An increased recovery rate and higher precision was obtained by using accelerated solvent extraction. This also increased cost-effectiveness: less time was needed for the clean-up procedures. Although the bioassay has been officially accepted by several organizations, sample clean-up methods may still be improved and simplified to improve accuracy, precision and sample throughput [97].

Another method to improve the output from the CALUX bioassay has been recently reported by Zhao *et al*. [109]. These authors improved the sensitivity of the CALUX bioassay by monitoring the modulators of the cell signaling pathways and modification of cell culture conditions. Their results showed that the addition of dexamethasone, which is a glucocorticoid receptor agonist, into the standard media enhanced the lower limits of detection and increased the magnitude of the response. Sato *et al*. [110] reported the successful use of a graphitic carbon solid-phase extraction method for water samples used for CALUX bioassay determination in comparison to chemical analysis using HRGC-HRMS.

Recently, Baston and Denison [103] reported that the potential of dioxin measurements by the CALUX bioassay could be improved via normalization of superinduction results. It was postulated that three factors determine the precision and accuracy of the CALUX bioassay: (1) the use of isolation procedures to obtain the desired compounds by using effective extraction and clean-up methods; (2) the use of appropriate criteria to establish a comparison of sample extracts and standard TCDD induction curves; and (3) the attempt to use data that cover the best possible range of induction in order to establish the optimal results having minimal and maximal plateaus and a slope comparable to the standard TCDD curve. According to these three elements, these authors emphasized that superinduction was observed even after the optimal clean-up method had been applied.

Overall it is clear that cell-based bioassays like CALUX are very suitable for screening of feed and food, but also water and soil, for the presence of dioxins and DL-PCBs. It is also clear that the clean-up based on acid silica is an important step not only to remove the fat but also many of the non-dioxin-like AhR-agonists and as such increases the selectivity of the test.

### 3.3. Sensor Technology

Sensor technology is nowadays gaining more interest as it is perceived to comprise several advantages over conventional chemical analysis and biological assays, for example, its simplicity, its cost-effectiveness and the possibility for real-time and on-site analysis. Physical and biological sensors (biosensors) are the two promising technologies that might be used for determination of dioxins and DL-PCBs throughout the food chain involving various types of specimens such as water, air, soil, feed, animal tissues and final products. In this review, the main focus lies on biosensors but we will also briefly discuss some physical sensors and combinations of both technologies.

#### 3.3.1. Physical Sensors

Even though this group of sensors has been successfully established and commercially applied for many industrial purposes, there is limited literature available that reports about the use of physical sensors for detecting dioxins and PCBs. Carbon nanotube technology offers the possibility for dioxin and PCB detection [111,112]. This technology might serve as a sensing element. Recently, the successful use of single wall carbon nanotube as a detection element for determining non-dioxin-like PCBs has been reported [111,112]. Other physical methods that are worth mentioning are nanostructured-based surface-enhanced Raman scattering spectroscopy [113–119], fluorescence quenching and enhancement [120] and the use of surface photo voltage [121] and porous anodic alumina (PAA) based capacitive sensors [122]. With these approaches PCB trace detection has been reported to be successfully established. However, so far, no physical sensor instrument for detection of dioxins and DL-PCBs in food and feed has been commercially developed.

#### 3.3.2. Biosensors

Currently, biosensors are interesting because of their advantages such as rapid, on-line or on-site analysis, minimal waste production, low cost of energy, less use of chemical reagents, miniaturization [123] and the possibility to combine assays using multiplex technologies [124]. As such they resemble bioassays in that they are effect-based but their application does not require a dedicated laboratory and less use of chemical reagents. In this review, we compared the requirements of biosensors for detecting dioxins and DL-PCBs along the food chain of milk, eggs and meat with the biosensors that are currently being used in medical and health care, and for environmental purposes, because they share similar requirements such as sensitivity, selectivity, limit of detection and stability. However, significant differences in matrices among food and food chain samples as compared to those encountered in the medical field, results in the need for additional preparation, extraction and clean-up procedures.

The definition of biosensor has been given by Thevenot *et al*. [125] as “a self-contained integrated device that is capable of providing specific quantitative or semi-quantitative analytical information using a biological recognition element (biochemical receptor), which is retained in direct spatial contact with a transduction element”. Biosensors can be distinguished from other bioanalytical systems, which would require additional processing steps, such as reagent addition. Biosensors consist of two main components: the biorecognition element, which recognizes the target contaminants, and the transducer, which converts the event into an electronic signal [126].

Theoretically, biosensors can be classified according to their biological recognition elements being used. These elements can be enzymes, antibodies, DNA, whole cells and other biological receptors. Some of these biorecognition elements have been studied for determining dioxins and PCBs.

##### Immunosensors

Immunosensors or antibody-based biosensors are very versatile [127]. Antibodies may be prepared as polyclonal, monoclonal or recombinant. The selection of antibodies depends on selective properties needed and the production method that is applied [126]. The best feature of this kind of sensor is its high selectivity for the selected antigens, in our case dioxins and DL-PCBs. The use of immunosensors for detection of non-dioxin-like PCBs in food samples has been successful [128–130]. However, some limitations have been established when this technique was applied to real samples, as they may incorrectly bind to other chemical compounds or contaminants that resemble the chemical structure of the antigen [131]. Laschi *et al*. [128] successfully developed a preliminary disposable electrochemical immunosensor for detection of non-dioxin-like PCBs in ruminant milk, adipose tissue and meat extracts. These authors used an electrochemical signal as a transducer. An accelerated solvent extractor (ASE) was used for sample extraction. Their results demonstrate that a higher sensitivity of the sensing element to the specific antigen (PCB 28) was observed compared to other congeners.

Centi *et al*. [129] used immunosensors for detecting PCBs in milk samples. By doing so, they demonstrated the sensitivity and reproducibility of the sensor. However, a highly efficient extraction technique (solid-phase extraction) was needed to acquire purified extracts to probe the sensor. In addition to the detection of PCBs in food samples, immunosensors using quartz crystal microbalance as a transducer to detect dioxins in fly ash samples, have also been successfully developed [131]. The sensor was developed for specifically binding to a TCDD derivative. The results showed that there was a high correlation to the golden standard: chemical analysis [131]. It was found that by this method, the lower detection limit was 1 part per trillion. However, extraction and clean-up procedures were still required prior to testing and these procedures play a major role in the accuracy and precision of the measurement.

##### Whole Cell-Based Biosensors

Whole cells or tissues can also be used as a sensing element in biosensors [132]. The CALUX method described above can be seen as a representative of a whole cell-based biosensor. Genetically engineered whole cell-based biosensors have been developed for direct detection of organophosphorus pesticides in water samples [133]. However, little information can be found in the scientific literature about the potential use for dioxins or PCBs. Recently, Gavlasova *et al*. [134] developed a whole cell-based biosensor for detecting PCB contamination in soil samples. These authors used *Pseudomonas* sp. P2 as biorecognition element based on optical detection. Technically, this microorganism can oxidize PCB molecules, which results in production of yellow *meta* ring-fission metabolites that can be measured through the absorption spectra by an optical transducer. This sensor can detect PCB contamination in extracts of soil samples. However, it failed to detect the contamination in non-extracted samples of soil [134]. In summary, the whole cell-based biosensor coupled with a simple extraction method, can be used as a screening test. The advantages of this type of biosensor for soil and sediment samples are its simple preparation and measurement techniques, which enables the use of low-cost instruments. However, limits of detection of these whole cell-based biosensors are above those required for feed and food samples and therefore need further development. An additional drawback found from the study of Gavlasova *et al.* was the presence of other yellow metabolites from unidentified factors which might hamper the accuracy and precision of the measurement.

##### Biomimetic Based Biosensors

A sensing element from this group was synthesized and designed to mimic a natural bioreceptor, such as antibody and enzyme [126], that can be used as a biorecognition element for sensor technology purposes. Antibodies that are used as sensor may denature when exposed to chemical reagents that are used during extraction and clean-up procedures. This may result in a reduction of the sensitivity of the assay. To solve this limitation, the use of synthetic peptides might be an alternative. Inuyama *et al*. [135] have developed an application for determining dioxins by the application of a dioxin-binding peptide as a detector for detecting contamination in soil samples. The sensitivity of this peptide sensing element can be improved by the use of on-bead technology. The concentration of dioxins was calculated using technology that measured fluorescence intensity, which reduced when the concentration of dioxins increased. A detection limit of 0.2 ng TCDD/mL was demonstrated. However, this still required extraction and clean-up procedures prior to evaluation.

Mascini *et al*. [136] also used a biomimetic approach combined with a quartz crystal microbalance piezoelectric transducer to determine dioxin and PCB contamination. Oligopeptides were synthesized and used to mimic the aryl hydrocarbon receptor (AhR) binding site and immobilized onto a gold surface. Their results demonstrate that the range of detection of TCDD, a dioxin mixture and PCBs was from 1 to 5 ppb, 1 to 10 ppb and 1 to 20 ppb, respectively with a coefficient of variation less than 15%. These authors applied this method for detecting dioxins in food products (e.g., chicken, eggs and milk) [120]. After sample extraction, two clean-up methods were compared: (1) acid/base liquid/liquid partitioning (simplification); and (2) acid/base silica, alumina and carbon. Their results demonstrated that a biomimetic receptor shows potential to detect dioxins and PCBs in different food matrices. In addition, clean-up methods did not give a different output. To date this method has not yet gained wide application.

Cytochrome c (Cyt c), a heme containing metalloprotein which is ubiquitious in a cellular context and involved in electron transfer processes, has been used as a biological recognition element to detect PCBs in aqueous solution by Hong *et al*. [137]. A conformation change of Cyt c, which is immobilized on a gold surface when exposed to PCB, was detected using surface plasmon resonance spectroscopy. These authors demonstrated that the detection limit of this method was as low as 0.1 ppb and needed only 10 min to complete the response.

## 4. Advantages, Limitations and Potentials of Biosensors for Detecting Dioxins and DL-PCBs along **the Food Chain**

The following criteria are generally used to assess the potential and limitations of sustainability indicators [138], and applied in this study to assess potential and limitations of methods available to detect contamination of samples with dioxins and DL-PCBs along the food chain of milk, eggs and meat: (1) validity, (2) simplicity, (3) sensitivity (4) relevance and (5) economic and technical feasibility.

- Validity: this criterion judges the potential of determination methods on dioxins and DL-PCBs in terms of accuracy (no or less bias, the measurements are close to their true values) and precision (high repeatability and conformity of measurements).
- Simplicity: this criterion focuses on the ease to use the selected determination method.
- Sensitivity: due to the ultra-low limits for dioxins and DL-PCBs in the food chain and related samples, highly sensitive recognition elements are needed to be able to qualify and/or quantify the contamination. This criterion is judged based on the potential of the detector for dioxins and DL-PCBs determination in terms of limits of detection.
- Relevance: this criterion is based on the interpretability of the output in relation to the biological toxicity.
- Economic and technical feasibility: this criterion is evaluated based on the cost-effectiveness and the possibility to use the method in a commercial context (eventually in the future).

Based on the above mentioned criteria, we assessed and scored each of the determination methods (Table 3).

Potential and limitations of HRGC-HRMS have been discussed elsewhere [13,91]. This method provides high sensitivity as well as highly accurate and precise output. Furthermore, the individual congeners, which are also important for identifying the source of the contamination, can be classified. Moreover, it is the only way to confirm the identity of the target compounds. Limitations of this analytical method are known. HRGC-HRMS is costly, due to the use of expensive standards, chemical reagents, machines and instruments with high maintenance cost. The need of well-trained personnel makes the HRGC-HRMS method complicated to run and to maintain. Low throughput is an additional drawback of chemical analysis. In addition, because of its specific contaminant determination, novel dioxin-like compounds e.g., polybrominated dioxins or the mixed bromo-chloro-dioxins will be overlooked.

The major advantages of physical sensors are high sensitivity and specificity. However, the outputs do not show the level of biological toxicity and physical sensors are still in the developmental phase.

Biosensors might be a promising technology for surveillance and monitoring the contamination of dioxins and DL-PCBs along the food chain because of their reliability, high throughputs and real-time determination. Timely, accurate and precise output can help decision-makers deciding the appropriate solutions for preventing contamination as an early warning system. These sensors are not yet applied in monitoring systems and still require further optimization and validation. Biosensors (in particular whole cell-based biosensors) usually reflect the biotoxicity of dioxins and DL-PCBs. The sensitivity of biosensors is lower compared with chemical analysis and bioassays. One of the advantages of biosensors is that they are primarily developed for ease of use. Within biosensor technology, whole cell-based biosensors present highest physiologically relevant output since they react to dioxins in a biologically relevant manner [139]. A major drawback of this group of biosensors is its slow response to the contaminants, which might be improved through novel genetic engineering.

Regarding the very low limits for the samples, potential interference by other compounds and the variety of sample matrices, some pre-treatment methods are still needed prior to determination by biosensors and this is likely to be a bottleneck for their development [140]. As a result, extraction methods using less sophisticated instruments and less hazardous chemical reagents can improve the potential of biosensors for detecting dioxins and DL-PCBs in food, feed and environmental samples.

## 5. Conclusions

This review provides insight into potential and limitations of different techniques for detecting presence of dioxins and DL-PCBs in the food chain of milk, eggs and meat. Since dioxins and DL-PCBs are highly toxic and ubiquitously present in food chain and its environment, efficient monitoring must be applied as an early warning system to prevent exposure of animals and humans.

The need of real-time and on-site determination of dioxins is of utmost importance in order to make correct and timely decisions. Chemical analysis is the gold standard method, but requires expensive and sophisticated facilities and instruments, costly reagents and well-trained operators. The CALUX bioassay is an established method, that can efficiently be used for screening of samples, even though extraction and clean-up methods are a prerequisite. Sensors are promising for detection of dioxins and DL-PCBs in the food chain, although they are still under development. Different biorecognition elements provide different advantages and limitations. Overall, immunosensors and biomimetic-based biosensors present advantages, such as low variation between producing batches and specificity to individual congeners of dioxins and DL-PCBs. Limitations are the lack of biological output and the inability to detect other dioxin-like contaminants. The susceptibility of immunosensors to residues of organic solvents, which are normally used in the step of sample extraction and clean-up, may reduce their potential when compared to biomimetic-based biosensors. Meanwhile, the whole cell-based biosensors show a high potential for detecting contamination by providing useful biological output. Several limitations of whole cell-based biosensors e.g., sensitivity, selectivity, limits of detection and user-friendly aspects need to be further optimized. In addition to further optimization, biosensor methodology requires further standardization in order to allow their application for excluding contaminant levels in food and feed above existing limits.

## Figures and Tables

**Table 1.** Maximum levels of dioxins and DL-PCBs in animal-source food (pg TEQ/g fat) set by the European Union [42].

**Food source**	**Maximum levels**	
	Sum of dioxins *	**Sum of dioxins + DL-PCBs**
Meat and meat products		
- Bovine animals and sheep	3.0	4.5
- Poultry	2.0	4.0
- Pigs	1.0	1.5
Raw milk and dairy products including butterfat	3.0	6.0
Hen eggs and egg products	3.0	6.0
Fat from following animals:		
- Bovine animals and sheep	3.0	4.5
- Poultry	2.0	4.0
- Pigs	1.0	1.5

**Table 2.** Estimated magnitudes of contamination sources in milk, egg and meat production systems.

**Components along the food chain of animal products**	**Milk production**		**Egg production**		**Meat production**	
	**Indoor^1^**	**Outdoor^2^**	**Indoor**	**Outdoor**	**Indoor**	**Outdoor**
Air (inhalation)	+	+	+	+	+	+
Soil	+	+++	0	+++	0	+++
Water	+	+	+	+	+	+
Worms and insects	0	0	0	++	0	+
Roughages ^3^	++	+++	0	+	++	+++
Domestic waste	0	0	0	+	0	0
Compound feed	+	+	++	++	+	+
Bedding material	+	+	+	+	+	+

1 refers to a production system without outdoor access (e.g., zero-grazing in milk production systems);

2 including organic, free range and outdoor production systems;

+++: high risk;

++: medium risk;

+: low risk; and 0: no risk;

3 Roughage in meat production systems is mainly provided to produce beef, not for pigs or poultry meat.

**Table 3.** Assessment of different determination methods for detection of dioxin and DL-PCB contamination along the food chain.

	**Validity**	**Simplicity**	**Sensitivity**	**Relevance**	Feasibility *
Chemical analysis (HRGC-HRMS)	+++	+	+++	+++	+++
Biological assay (CALUX)	++	++	+++	+++	+++
Sensor technology					
- Physical sensors	+	++	+	+	+
- Biosensors					
- Immunosensors	+	++	+	++	++
- Whole cell-based biosensors	+	++	+	+++	+
- Biomimetic-based biosensors	+	++	+	++	+

+++ high potential,

++ medium potential and

+ low potential;

* only technical feasibility is assessed. Economic feasibility (e.g., cost-effectiveness) of sensors cannot be assessed as, so far, there are no sensors for detecting dioxins and DL-PCBs available on a commercial scale.

## References

[1] Kulkarni P.S., Crespo J.G., Afonso C.A.M. (2008). Dioxins sources and current remediation technologies—A review. Environ. Int.

[2] Durand B., Dufour B., Fraisse D., Defour S., Duhem K., Le-Barillec K. (2008). Levels of PCDDs, PCDFs and dioxin-like PCBs in raw cow’s milk collected in France in 2006. Chemosphere.

[3] Kulkarni P.S., Crespo J.G., Afonso C.A.M., Jerome O.N. (2011). Dioxins. Encyclopedia of Environmental Health.

[4] Schecter A., Birnbaum L., Ryan J.J., Constable J.D. (2006). Dioxins: An overview. Environ. Res.

[5] Schecter A.J., Colacino J.A., Birnbaum L.S., Jerome O.N. (2011). Dioxins: health effects. Encyclopedia of Environmental Health.

[6] van den Berg M., Birnbaum L.S., Denison M., de Vito M., Farland W., Feeley M., Fiedler H., Hakansson H., Hanberg A., Haws L., Rose M., Safe S., Schrenk D., Tohyama C., Tritscher A., Tuomisto J., Tysklind M., Walker N., Peterson R.E. (2006). The 2005 World Health Organization reevaluation of human and mammalian toxic equivalency factors for dioxins and dioxin-like compounds. Toxicol. Sci.

[7] Ahmed F.E. (2003). Analysis of polychlorinated biphenyls in food products. TrAC Trends Anal. Chem.

[8] Thomas G.O., Sven Erik J., Brian F. (2008). Polychlorinated biphenyls. Encyclopedia of Ecology.

[9] Heres L., Hoogenboom R., Herbes R., Traag W., Urlings B. (2010). Tracing and analytical results of the dioxin contamination incident in 2008 originating from the Republic of Ireland. Food Addit. Contam. Part A Chem.

[10] Kennedy J., Delaney L., Hudson E.M., McGloin A., Wall P.G. (2010). Public perceptions of the dioxin incident in Irish pork. J. Risk Res.

[11] Kupferschmidt K. (2011). Dioxin scandal triggers food debate in Germany. Can. Med. Assoc. J.

[12] Alcoser V.H.L., Velthuis A.G.J., Hoogenboom L.A.P., van der Fels-Klerx H.J. (2011). Financial impact of a dioxin incident in the dutch dairy chain. J. Food Prot.

[13] Reiner E.J. (2010). The analysis of dioxins and related compounds. Mass Spectrom. Rev.

[14] Hoogenboom L., Goeyens L., Carbonnelle S., van Loco J., Beernaert H., Baeyens W., Traag W., Bovee T., Jacobs G., Schoeters G. (2006). The CALUX bioassay: Current status of its application to screening food and feed. TrAC Trends Anal. Chem.

[15] Justino C.I.L., Rocha-Santos T.A., Duarte A.C. (2010). Review of analytical figures of merit of sensors and biosensors in clinical applications. TrAC Trends Anal. Chem.

[16] Nien P.-C., Tung T.-S., Ho K.-C. (2006). Amperometric glucose biosensor based on entrapment of glucose oxidase in a poly(3,4-ethylenedioxythiophene) film. Electroanalysis.

[17] Tan X., Capar G., Li K. (2005). Analysis of dissolved oxygen removal in hollow fibre membrane modules: Effect of water vapour. J. Membr. Sci.

[18] Ponomareva O.N., Arlyapov V.A., Alferov V.A., Reshetilov A.N. (2011). Microbial biosensors for detection of biological oxygen demand (a review). Appl. Biochem. Microbiol.

[19] Palchetti I., Mascini M. (2008). Nucleic acid biosensors for environmental pollution monitoring. Analyst.

[20] Wanekaya A.K., Chen W., Mulchandani A. (2008). Recent biosensing developments in environmental security. J. Environ. Monit.

[21] González-Martínez M., Puchades R., Maquieira A. (2007). Optical immunosensors for environmental monitoring: How far have we come?. Anal. Bioanal. Chem.

[22] Kärkkäinen R.M., Drasbek M.R., McDowall I., Smith C.J., Young N.W.G., Bonwick G.A. (2011). Aptamers for safety and quality assurance in the food industry: Detection of pathogens. Int. J. Food Sci. Technol.

[23] Nugen S.R., Baeumner A.J. (2008). Trends and opportunities in food pathogen detection. Anal. Bioanal. Chem.

[24] Petz M. (2009). Recent applications of surface plasmon resonance biosensors for analyzing residues and contaminants in food. Monatshefte Chem.

[25] Eijkelkamp J., Aarts H., Fels-Klerx H. (2009). Suitability of rapid detection methods for salmonella in poultry slaughterhouses. Food Anal. Methods.

[26] Ahlborg U.G., Becking G.C., Birnbaum L.S., Brouwer A., Derks H., Feeley M., Golor G., Hanberg A., Larsen J.C., Liem A.K.D., Safe S.H., Schlatter C., Waern F., Younes M., Yrjänheikki E. (1994). Toxic equivalency factors for dioxin-like PCBs: Report on WHO-ECEH and IPCS consultation, December 1993. Chemosphere.

[27] van den Berg M., de Jongh J., Poiger H., Olson J.R. (1994). The toxicokinetics and metabolism of polychlorinated dibenzo-p-dioxins (PCDDS) and dibenzofurans (PCDFS) and their relevance for toxicity. Crit. Rev. Toxicol.

[28] NRC Health Risks from Dioxin and Related Compound: Evaluation of the EPA Reassessment.

[29] Safe S.H. (1986). Comparative toxicology and mechanism of action of polychlorinated dibenzo-p-dioxins and dibenzofurans. Annu. Rev. Pharmacol. Toxicol.

[30] Marinkovic N., Pasalic D., Ferencak G., Grskovic B., Rukavina A.S. (2010). Dioxins and human toxicity. Arh. Hig. Rada Toksikol.

[31] Foster W.G., Maharaj-Briceno S., Cyr D.G. (2011). Dioxin-induced changes in epididymal sperm count and spermatogenesis. Cienc. Saude Coletiva.

[32] Balabanič D., Rupnik M., Klemenčič A.K. (2011). Negative impact of endocrine-disrupting compounds on human reproductive health. Reprod. Fertil. Dev.

[33] Longnecker M.P., Rogan W.J., Lucier G. (1997). The human health effects of DDT (dichlorodiphenyl-trichloroethane) and PCBS (polychlorinated biphenyls) and an overview of organochlorines in public health. Annu. Rev. Public Health.

[34] Okey A.B. (2007). Special contribution—An aryl hydrocarbon receptor odyssey to the shores of toxicology: The deichmann lecture, international congress of toxicology-XI. Toxicol. Sci.

[35] de Waard W.J., Aarts J.M.M.J.G., Peijnenburg A.C.M., de Kok T.M.C.M., van Schooten F.J., Hoogenboom L.A.P. (2008). Ah receptor agonist activity in frequently consumed food items. Food Addit. Contam. Part A.

[36] van der Heiden E., Bechoux N., Muller M., Sergent T., Schneider Y.-J., Larondelle Y., Maghuin-Rogister G., Scippo M.-L. (2009). Food flavonoid aryl hydrocarbon receptor-mediated agonistic/antagonistic/synergic activities in human and rat reporter gene assays. Anal. Chim. Acta.

[37] Zhang S., Qin C., Safe S.H. (2003). Flavonoids as aryl hydrocarbon receptor agonists/antagonists: Effects of structure and cell context. Environ. Health Perspect.

[38] Denison M.S., Pandini A., Nagy S.R., Baldwin E.P., Bonati L. (2002). Ligand binding and activation of the Ah receptor. Chem. Biol. Interact.

[39] Bohonowych J.E., Denison M.S. (2007). Persistent binding of ligands to the aryl hydrocarbon receptor. Toxicol. Sci.

[40] de Mul A., Bakker M.I., Zeilmaker M.J., Traag W.A., van Leeuwen S.P.J., Hoogenboom R., Boon P.E., van Klaveren J.D. (2008). Dietary exposure to dioxins and dioxin-like PCBs in The Netherlands anno 2004. Regul. Toxicol. Pharmacol.

[41] Franzblau A., Hedgeman E., Jolliet O., Knutson K., Towey T., Chen Q., Hong B., Adriaens P., Demond A., Garabrant D.H., Gillespie B.W., Lepkowski J. (2010). Case report: The University of Michigan dioxin exposure study: A follow-up investigation of a case with high serum concentration of 2,3,4,7,8-pentachlorodibenzofuran. Environ. Health Perspect.

[42] (2006). Commission regulation (EC) No 1881/2006 of 19 December setting maximum levels for certain contaminants in foodstuffs. OJEC.

[43] Sorg O., Zennegg M., Schmid P., Fedosyuk R., Valikhnovskyi R., Gaide O., Kniazevych V., Saurat J.H. (2009). 2,3,7,8-Tetrachlorodibenzo-p-dioxin (TCDD) poisoning in Victor Yushchenko: Identification and measurement of TCDD metabolites. Lancet.

[44] Hakk H., Diliberto J.J., Birnbaum L.S. (2009). The effect of dose on 2,3,7,8-TCDD tissue distribution, metabolism and elimination in CYP1A2 (−/−) knockout and C57BL/6N parental strains of mice. Toxicol. Appl. Pharmacol.

[45] Geyer H.J., Schramm K.-W., Anton Feicht E., Behechti A., Steinberg C., Brüggemann R., Poiger H., Henkelmann B., Kettrup A. (2002). Half-lives of tetra-, penta-, hexa-, hepta-, and octachlorodibenzo-p-dioxin in rats, monkeys, and humans—A critical review. Chemosphere.

[46] Jackson J.A., Birnbaum L.S., Diliberto J.J. (1998). Effects of age, sex, and pharmacologic agents on the biliary elimination of 2,3,7,8-tetrachlorodibenzo-p-dioxin (TCDD) in F344 rats. Drug Metab. Dispos.

[47] Fries G.F., Paustenbach D.J., Mather D.B., Luksemburg W.J. (1999). A congener specific evaluation of transfer of chlorinated dibenzo-p-dioxins and dibenzofurans to milk of cows following ingestion of pentachlorophenol-treated wood. Environ. Sci. Technol.

[48] Adekunte A.O., Tiwari B.K., O’Donnell C.P. (2010). Exposure assessment of dioxins and dioxin-like PCBs in pasteurised bovine milk using probabilistic modelling. Chemosphere.

[49] Hoogenboom L.A.P., Kan C.A., Zeilmaker M.J., van Eijkeren J., Traag W.A. (2006). Carry-over of dioxins and PCBs from feed and soil to eggs at low contamination levels—Influence of mycotoxin binders on the carry-over from feed to eggs. Food Addit. Contam.

[50] Kijlstra A., Meerburg B.G., Bos A.P. (2009). Food safety in free-range and organic livestock systems: Risk management and responsibility. J. Food Prot.

[51] de Vries M., Kwakkel R.P., Kijlstra A. (2006). Dioxins in organic eggs: A review. NJAS-Wagen. J. Life Sci.

[52] Holt P.S., Davies R.H., Dewulf J., Gast R.K., Huwe J.K., Jones D.R., Waltman D., Willian K.R. (2011). The impact of different housing systems on egg safety and quality. Poult. Sci.

[53] Kijlstra A., Traag W.A., Hoogenboom L.A.P. (2007). Effect of flock size on dioxin levels in eggs from chickens kept outside. Poult. Sci.

[54] Rychen G., Jurjanz S., Toussaint H., Feidt C. (2008). Dairy ruminant exposure to persistent organic pollutants and excretion to milk. Animal.

[55] Schoeters G., Hoogenboom R. (2006). Contamination of free-range chicken eggs with dioxins and dioxin-like polychlorinated biphenyls. Mol. Nutr. Food Res.

[56] van Overmeire I., Pussemier L., Hanot V., de Temmerman L., Hoenig M., Goeyens L. (2006). Chemical contamination of free-range eggs from Belgium. Food Addit. Contam.

[57] Schwind K.H., Danicke S., Jira W. (2010). Survey of dioxins, dioxin-like PCBs and marker PCBs in German feeds of plant origin. J. Verbrauch. Lebensm.

[58] Boxall A.B.A., Hardy A., Beulke S., Boucard T., Burgin L., Falloon P.D., Haygarth P.M., Hutchinson T., Kovats R.S., Leonardi G., Levy L.S., Nichols G., Parsons S.A., Potts L., Stone D., Topp E., Turley D.B., Walsh K., Wellington E.M.H., Williams R.J. (2009). Impacts of climate change on indirect human exposure to pathogens and chemicals from agriculture. Environ. Health Perspect.

[59] Thomson B., Rose M. (2011). Environmental contaminants in foods and feeds in the light of climate change. Qual. Assur. Saf. Crop. Foods.

[60] Kim E.J., Oh J.E., Chang Y.S. (2003). Effects of forest fire on the level and distribution of PCDD/Fs and PAHs in soil. Sci. Total Environ.

[61] Black R.R., Meyer C.P., Touati A., Gullett B.K., Fiedler H., Mueller J.F. (2011). Emissions of PCDD and PCDF from combustion of forest fuels and sugarcane: A comparison between field measurements and simulations in a laboratory burn facility. Chemosphere.

[62] Hsu J.F., Guo H.R., Wang H.W., Liao C.K., Liao P.C. (2011). An occupational exposure assessment of polychlorinated dibenzo-p-dioxin and dibenzofurans in firefighters. Chemosphere.

[63] Gullett B., Touati A., Oudejans L. (2008). PCDD/F and aromatic emissions from simulated forest and grassland fires. Atmos. Environ.

[64] Estrellan C.R., Iino F. (2010). Toxic emissions from open burning. Chemosphere.

[65] Vassiliadou I., Papadopoulos A., Costopoulou D., Vasiliadou S., Christoforou S., Leondiadis L. (2009). Dioxin contamination after an accidental fire in the municipal landfill of Tagarades, Thessaloniki, Greece. Chemosphere.

[66] Oh J.E., Choi S.D., Lee S.J., Chang Y.S. (2006). Influence of a municipal solid waste incinerator on ambient air and soil PCDD/Fs levels. Chemosphere.

[67] Menotta S., D’Antonio M., Diegoli G., Montella L., Raccanelli S., Fedrizzi G. (2010). Depletion study of PCDD/Fs and dioxin-like PCBs concentrations in contaminated home-produced eggs: Preliminary study. Anal. Chim. Acta.

[68] Sapkota A.R., Lefferts L.Y., McKenzie S., Walker P. (2007). What do we feed to food-production animals? A review of animal feed ingredients and their potential impacts on human health. Environ. Health Perspect.

[69] Hoogenboom R., Zeilmaker M., van Eijkeren J., Kan K., Mengelers M., Luykx D., Traag W. (2010). Kaolinic clay derived PCDD/Fs in the feed chain from a sorting process for potatoes. Chemosphere.

[70] Turrio-Baldassarri L., Alivernini S., Carasi S., Casella M., Fuselli S., Iacovella N., Iamiceli A.L., La Rocca C., Scarcella C., Battistelli C.L. (2009). PCB, PCDD and PCDF contamination of food of animal origin as the effect of soil pollution and the cause of human exposure in Brescia. Chemosphere.

[71] Perry T.W., Everson R.J., Hendrix K.S., Peterson R.C., Weinland K.M., Robinson F.R. (1981). Dietary Aroclor 1254 in the milk fat of lactating beef cattle. J. Diary Sci.

[72] Brambilla G., Fochi I., Falce M., de Filippis S.P., Ubaldi A., di Domenico A. (2008). PCDD and PCDF depletion in milk from dairy cows according to the herd metabolic scenario. Chemosphere.

[73] Hsu J.F., Chen C., Liao P.C. (2010). Elevated PCDD/F levels and distinctive PCDD/F congener profiles in free range eggs. J. Agric. Food Chem.

[74] van Overmeire I., Pussemier L., Waegeneers N., Hanot V., Windal I., Boxus L., Covaci A., Eppe G., Scippo M.L., Sioen I., Bilau M., Gellynck X., de Steur H., Tangni E.K., Goeyens L. (2009). Assessment of the chemical contamination in home-produced eggs in Belgium: General overview of the CONTEGG study. Sci. Total Environ.

[75] van Overmeire I., Waegeneers N., Sioen I., Bilau M., de Henauw S., Goeyens L., Pussemier L., Eppe G. (2009). PCDD/Fs and dioxin-like PCBs in home-produced eggs from Belgium: Levels, contamination sources and health risks. Sci. Total Environ.

[76] Asari M., Takatsuki H., Yamazaki M., Azuma T., Takigami H., Sakai S. (2004). Waste wood recycling as animal bedding and development of bio-monitoring tool using the CALUX assay. Environ. Int.

[77] Brambilla G., Fochi I., de Filippis S.P., Iacovella N., di Domenico A. (2009). Pentachlorophenol, polychlorodibenzodioxin and polychlorodibenzofuran in eggs from hens exposed to contaminated wood shavings. Food Addit. Contam. Part A Chem.

[78] Fries G.F., Feil V.J., Zaylskie R.G., Bialek K.M., Rice C.P. (2002). Treated wood in livestock facilities: Relationships among residues of pentachlorophenol, dioxins, and furans in wood and beef. Environ. Pollut.

[79] Fernandes A.R., Foxall C., Lovett A., Rose M., Dowding A. (2011). The assimilation of dioxins and PCBs in conventionally reared farm animals: Occurrence and biotransfer factors. Chemosphere.

[80] Huwe J., Pagan-Rodriguez D., Abdelmajid N., Clinch N., Gordon D., Holterman J., Zaki E., Lorentzsen M., Dearfield K. (2009). Survey of polychlorinated dibenzo-p-dioxins, polychlorinated dibenzofurans, and non-ortho-polychlorinated biphenyls in US meat and poultry, 2007–2008: Effect of new toxic equivalency factors on toxic equivalency levels, patterns, and temporal trends. J. Agric. Food Chem.

[81] Hess H.D., Geinoz M. (2011). A farm survey on the presence of dioxins and dl-PCB in beef production systems in Switzerland. Biotechnol. Agron. Soc. Environ.

[82] Brambilla G., de Filippis S.P., Iamiceli A.L., Iacovella N., Abate V., Aronica V., di Marco V., di Domenico A. (2011). Bioaccumulation of dioxin-like substances and selected brominated flame retardant congeners in the fat and livers of black pigs farmed within the Nebrodi Regional Park of Sicily. J. Food Prot.

[83] Dujardin M., Narbonne J.F., Alexander S. (2000). The Belgian dioxin crisis. Biomed. Res.

[84] Kim M., Kim D.G., Choi S.W., Guerrero P., Norambuena J., Chung G.S. (2011). Formation of polychlorinated dibenzo-p-dioxins/dibenzofurans (PCDD/Fs) from a refinery process for zinc oxide used in feed additives: A source of dioxin contamination in Chilean pork. Chemosphere.

[85] Hoogenboom R., Bovee T., Portier L., Bor G., van der Weg G., Onstenk C., Traag W. (2004). The German bakery waste incident; Use of a combined approach of screening and confirmation for dioxins in feed and food. Talanta.

[86] Furst P. (2011). Dioxins in feed and food again—Real or perceived risk?. Eur. J. Lipid Sci. Technol.

[87] (2011). European Union acts on dioxin contamination of food. TrAC Trends Anal. Chem.

[88] Spitaler M., Iben C., Tausch H. (2005). Dioxin residues in the edible tissue of finishing pigs after dioxin feeding. J. Anim. Physiol. Anim. Nutr.

[89] Hoogenboom L.A.P., Kan C.A., Bovee T.F.H., van der Weg G., Onstenk C., Traag W.A. (2004). Residues of dioxins and PCBs in fat of growing pigs and broilers fed contaminated feed. Chemosphere.

[90] United Staates of Environmental Protection Agency (EPA) (1994). Method 1613, Revision B, Tetra-through Octa-Chlorinated Dioxins and Furans by Isotope Dilution HRGC/HRMS.

[91] Reiner E.J., Clement R.E., Okey A.B., Marvin C.H. (2006). Advances in analytical techniques for polychlorinated dibenzo-p-dioxins, polychlorinated dibenzofurans and dioxin-like PCBs. Anal. Bioanal. Chem.

[92] Zabelina O.N., Saloutin V.I., Chupakhin O.N. (2010). Analysis of polychlorinated biphenyl mixtures by gas chromatography. J. Anal. Chem.

[93] Wang B., Yu G., Zhang T.T., Huang J., Wang T., Nakamura M., Handa H., Huang C.C., Murata H. (2009). CALUX bioassay of dioxin-like compounds in sediments from the Haihe river, China. Soil. Sediment. Contam.

[94] Safe S., Wormke M. (2003). Inhibitory aryl hydrocarbon receptor—estrogen receptor α cross-talk and mechanisms of action. Chem. Res. Toxicol.

[95] Kennedy S.W., Lorenzen A., James C.A., Collins B.T. (1993). Ethoxyresorufin-O-deethylase and porphyrin analysis in chicken embryo hepatocyte cultures with a fluorescence multiwell plate reader. Anal. Biochem.

[96] Garrison P.M., Tullis K., Aarts J.M.M.J.G., Brouwer A., Giesy J.P., Denison M.S. (1996). Species-specific recombinant cell lines as bioassay systems for the detection of 2,3,7,8-tetrachlorodibenzo-p-dioxin-like chemicals. Fundam. Appl. Toxicol.

[97] Gizzi G., Hoogenboom L.A.P., von Holst C., Rose M., Anklam E. (2005). Determination of dioxins (PCDDs/PCDFs) and PCBs in food and feed using the DR CALUX (R) bioassay: Results of an international validation study. Food Addit. Contam.

[98] Hoogenboom R., Bovee T., Traag W., Hoogerbrugge R., Baumann B., Portier L., van de Weg G., de Vries J. (2006). The use of the DR CALUX (R) bioassay and indicator polychlorinated biphenyls for screening of elevated levels of dioxins and dioxin-like polychlorinated biphenyls in eel. Mol. Nutr. Food Res.

[99] van Leeuwen S.P.J., Leonards P.E.G., Traag W.A., Hoogenboom L.A.P., de Boer J. (2007). Polychlorinated dibenzo-p-dioxins, dibenzofurans and biphenyls in fish from the Netherlands: Concentrations, profiles and comparison with DR CALUX (R) bioassay results. Anal. Bioanal. Chem.

[100] Bovee T.F.H., Hoogenboom L.A.P., Hamers A.R.M., Traag W.A., Zuidema T., Aarts J., Brouwer A., Kuiper H.A. (1998). Validation and use of the CALUX-bioassay for the determination of dioxins and PCBs in bovine milk. Food Addit. Contam.

[101] Malisch R. (2000). Increase of the PCDD/F-contamination of milk, butter and meat samples by use of contaminated citrus pulp. Chemosphere.

[102] Bernard A., Hermans C., Broeckaert F., de Poorter G., de Cock A., Houins G. (1999). Food contamination by PCBs and dioxins. Nature.

[103] Baston D.S., Denison M.S. (2011). Considerations for potency equivalent calculations in the Ah receptor-based CALUX bioassay: Normalization of superinduction results for improved sample potency estimation. Talanta.

[104] Amakura Y., Tsutsumi T., Nakamura M., Handa H., Yoshimura M., Matsuda R., Yoshida T. (2011). Aryl hydrocarbon receptor ligand activity of commercial health foods. Food Chem.

[105] Windal I., Denison M.S., Birnbaum L.S., van Wouwe N., Baeyens W., Goeyens L. (2005). Chemically activated luciferase gene expression (CALUX) cell bioassay analysis for the estimation of dioxin-like activity: Critical parameters of the CALUX procedure that impact assay results. Environ. Sci. Technol.

[106] Jeong S.H., Cho J.H., Park J.M., Denison M.S. (2005). Rapid bioassay for the determination of dioxins and dioxin-like PCDFs and PCBs in meat and animal feeds. J. Anal. Toxicol.

[107] Chao H.R., Wang Y.F., Lin D.Y., Cheng Y.T., Tsou T.C. (2011). Fast cleanup system combined with a dioxin-responsive element-driven luciferase bioassay for analysis of polychlorinated dibenzo-p-dioxins/furans in sediments and soils. Bull. Environ. Contam. Toxicol.

[108] Stypula-Trebas S., Piskorska-Pliszczynska J. (2010). Comparison of extraction and cleanup procedures for the determination of PCDD/Ffs in feed samples with the calux bioassay. Bull. Vet. Inst. Pulawy.

[109] Zhao B., Baston D.S., Khan E., Sorrentino C., Denison M.S. (2010). Enhancing the response of CALUX and CAFLUX cell bioassays for quantitative detection of dioxin-like compounds. Sci. China Chem.

[110] Sato M., Takigami H., Hayakawa K., Sakai S. (2010). Water-quality monitoring technique for dioxins during dredging using on-site solid phase extraction with graphitic carbon and analysis with DR-CALUX. J. Environ. Sci. Health Part A Toxichazard. Subst. Environ. Eng.

[111] Wei Y., Kong L.T., Yang R., Wang L., Liu J.H., Huang X.J. (2011). Electrochemical impedance determination of polychlorinated biphenyl using a pyrenecyclodextrin-decorated single-walled carbon nanotube hybrid. Chem. Commun.

[112] Fagan S.B., Santos E.J.G., Souza A.G., Mendes J., Fazzio A. (2007). Ab initio study of 2,3,7,8-tetrachlorinated dibenzo-p-dioxin adsorption on single wall carbon nanotubes. Chem. Phys. Lett.

[113] Abalde-Cela S., Ho S., Rodríguez-González B., Correa-Duarte M.A., Álvarez-Puebla R.A., Liz-Marzán L.M., Kotov N.A. (2009). Loading of exponentially grown LBL films with silver nanoparticles and their application to generalized SERS detection. Angew. Chem.

[114] Yang Y., Meng G. (2010). Ag dendritic nanostructures for rapid detection of polychlorinated biphenyls based on surface-enhanced Raman scattering effect. J. Appl. Phys.

[115] Bantz K.C., Haynes C.L. (2009). Surface-enhanced Raman scattering detection and discrimination of polychlorinated biphenyls. Vib. Spectrosc.

[116] Huang Z., Meng G., Huang Q., Yang Y., Zhu C., Tang C. (2010). Improved SERS performance from Au nanopillar arrays by abridging the pillar tip spacing by Ag sputtering. Adv. Mater.

[117] Zhu C., Meng G., Huang Q., Huang Z., Chu Z. (2011). Au hierarchical micro/nanotower arrays and their improved SERS effect by Ag nanoparticle decoration. Cryst. Growth Des.

[118] Zhu C., Meng G., Huang Q., Zhang Z., Xu Q., Liu G., Huang Z., Chu Z. (2011). Ag nanosheet-assembled micro-hemispheres as effective SERS substrates. Chem. Commun.

[119] Zhu C., Meng G., Huang Q., Huang Z. (2011). Vertically aligned Ag nanoplate-assembled film as a sensitive and reproducible SERS substrate for the detection of PCB-77. J. Hazard. Mater.

[120] Wang M., Meng G., Huang Q., Li M., Li Z., Tang C. (2011). Fluorescence detection of trace PCB101 based on PITC immobilized on porous AAO membrane. Analyst.

[121] Li M., Meng G., Huang Q., Yin Z., Wu M., Zhang Z., Kong M. (2010). Prototype of a porous ZnO SPV-based sensor for PCB detection at room temperature under visible light illumination. Langmuir.

[122] Jin Z., Meng F., Liu J., Li M., Kong L., Liu J. (2011). A novel porous anodic alumina based capacitive sensor towards trace detection of PCBs. Sens. Actuat. B Chem.

[123] Farre M., Perez S., Goncalves C., Alpendurada M.F., Barcelo D. (2010). Green analytical chemistry in the determination of organic pollutants in the aquatic environment. TrAC Trends Anal. Chem.

[124] Rebe Raz S., Haasnoot W. (2011). Multiplex bioanalytical methods for food and environmental monitoring. TrAC Trends Anal. Chem.

[125] Thevenot D.R., Toth K., Durst R.A., Wilson G.S. (2001). Electrochemical biosensors: Recommended definitions and classification. Biosens. Bioelectron.

[126] Velusamy V., Arshak K., Korostynska O., Oliwa K., Adley C. (2010). An overview of foodborne pathogen detection: In the perspective of biosensors. Biotechnol. Adv.

[127] Rogers K.R. (2006). Recent advances in biosensor techniques for environmental monitoring. Anal. Chim. Acta.

[128] Laschi S., Mascini M., Scortichini G., Fránek M., Mascini M. (2003). Polychlorinated biphenyls (PCBs) detection in food samples using an electrochemical immunosensor. J. Agric. Food Chem.

[129] Centi S., Silva E., Laschi S., Palchetti I., Mascini M. (2007). Polychlorinated biphenyls (PCBs) detection in milk samples by an electrochemical magneto-immunosensor (EMI) coupled to solid-phase extraction (SPE) and disposable low-density arrays. Anal. Chim. Acta.

[130] Silva E., Mascini M., Centi S., Turner A.P.F. (2007). Detection of polychlorinated biphenyls (PCBs) in milk using a disposable immunomagnetic electrochemical sensor. Anal. Lett.

[131] Kurosawa S., Aizawa H., Park J.W. (2005). Quartz crystal microbalance immunosensor for highly sensitive 2,3,7,8-tetrachlorodibenzo-p-dioxin detection in fly ash from municipal solid waste incinerators. Analyst.

[132] Su L.A., Jia W.Z., Hou C.J., Lei Y. (2011). Microbial biosensors: A review. Biosens. Bioelectron.

[133] Lei Y., Mulchandani P., Chen W., Mulchandani A. (2007). Biosensor for direct determination of fenitrothion and EPN using recombinant Pseudomonas putida JS444 with surface-expressed organophosphorous hydrolase. 2. Modified carbon paste electrode. Appl. Biochem. Biotechnol.

[134] Gavlasova P., Kuncova G., Kochankova L., Mackova M. (2008). Whole cell biosensor for polychlorinated biphenyl analysis based on optical detection. Int. Biodeterior. Biodegrad.

[135] Inuyama Y., Nakamura C., Oka T., Yoneda Y., Obataya I., Santo N., Miyake J. (2007). Simple and high-sensitivity detection of dioxin using dioxin-binding pentapeptide. Biosens. Bioelectron.

[136] Mascini M., Macagnano A., Monti D., del Carlo M., Paolesse R., Chen B., Warner P., D’Amico A., di Natale C., Compagnone D. (2004). Piezoelectric sensors for dioxins: A biomimetic approach. Biosens. Bioelectron.

[137] Kim M., Kim S., Yun S.J., Kwon J.W., Son S.W. (2007). Evaluation of PCDD/Fs characterization in animal feed and feed additives. Chemosphere.

[138] Mollenhorst H., Berentsen P.B.M., de Boer I.J.M. (2006). On-farm quantification of sustainability indicators: An application to egg production systems. Br. Poult. Sci.

[139] Scott D., Dikici E., Ensor M., Daunert S. (2011). Bioluminescence and its impact on bioanalysis. Annu. Rev. Anal. Chem.

[140] Sanvicens N., Mannelli I., Salvador J.P., Valera E., Marco M.P. (2011). Biosensors for pharmaceuticals based on novel technology. TrAC Trends Anal. Chem.

